# The genome of the coral model sea anemone *Exaiptasia diaphana *(Aiptasia) strain F003

**DOI:** 10.46471/gigabyte.188

**Published:** 2026-09-14

**Authors:** Melanie Dörr, Abdoallah Sharaf, Luigi Colin, Kevin Schuster, Alyssa C. Bell, Christian R. Voolstra

**Affiliations:** ^1^ Department of Biology, University of Konstanz, Konstanz, Germany; ^2^ SequAna Core Facility, Department of Biology, University of Konstanz, Konstanz, Germany

## Abstract

We present a genome assembly of Aiptasia strain F003, a broadly used laboratory strain of the sea anemone and coral model *Exaiptasia diaphana* (Cnidaria; Anthozoa; Hexacorallia; Actiniaria; Aiptasiidae; *Exaiptasia*). The genome assembly spans 237.34 Mb across 12,480 contigs with a contig N50 of 76.47 kb (12,423 scaffolds with a scaffold N50 of 77.93 kb), including a single-contig mitochondrial genome with a length of 19.79 kb. The assembly is highly complete with a BUSCO completeness of 96.50% based on the metazoa dataset, comprising 94.80% single-copy and 1.70% duplicated BUSCO genes; 1.70% were fragmented and 1.80% were missing. Genome annotation identified 29,589 protein-coding genes (including 2 pseudogenes) and a repeat content of 32.89%. The genome of the female Aiptasia strain F003 enhances the utility of a key cnidarian model organism by enabling comparisons among Aiptasia strains in studies of symbiosis, microbiomes, and thermal stress. It thereby strengthens the value of Aiptasia as a model for investigating the mechanisms underlying coral holobiont function, response, and resilience to environmental change.

## Introduction

**Figure 1.**
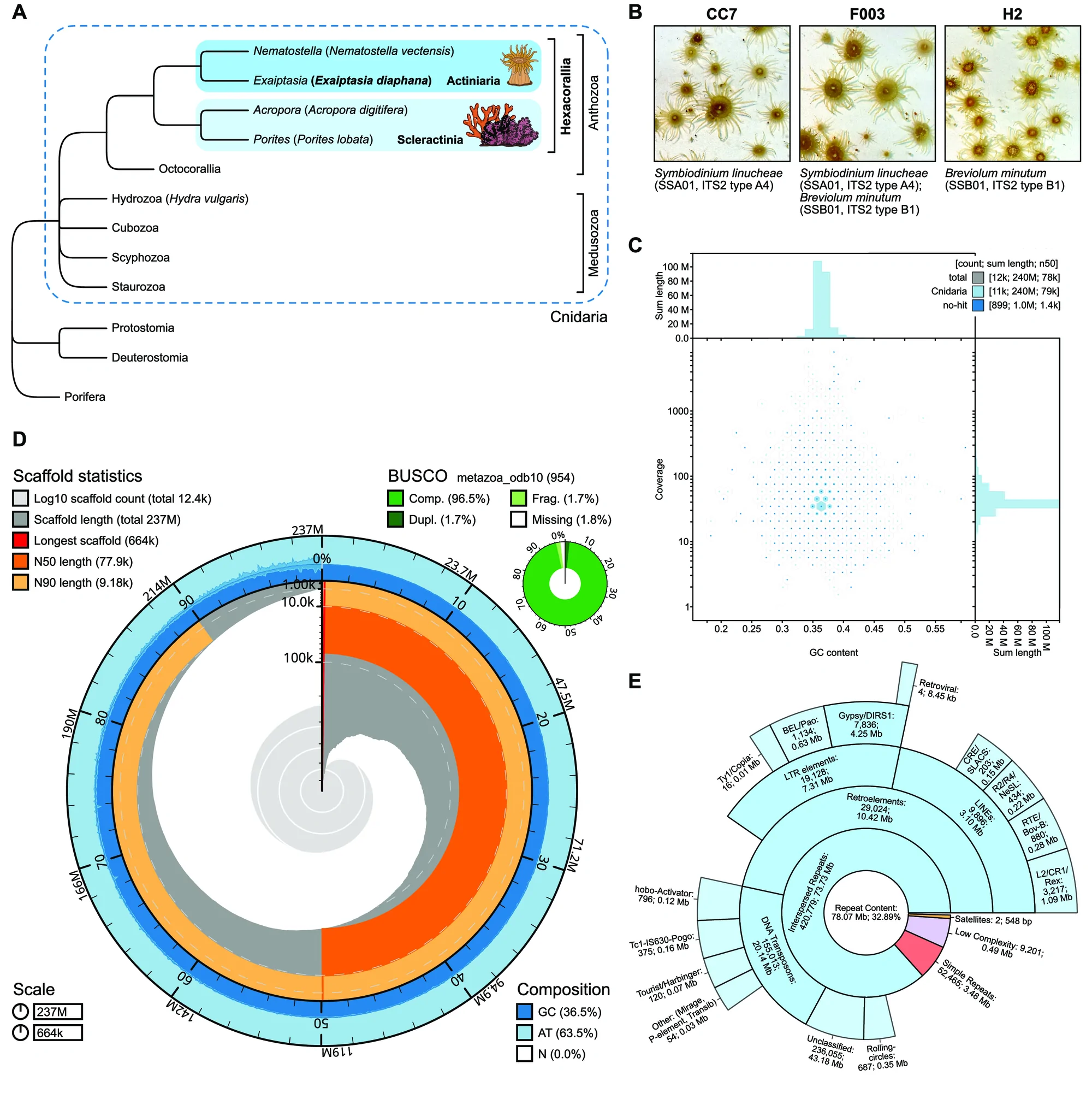
The genome of *Exaiptasia diaphana *(Aiptasia) strain F003. (A) Cladogram depicting the placement of *Exaiptasia diaphana *within the phylum Cnidaria. Actinitarian sea anemones are grouped together with sessile cnidarians, notably stony corals (Scleractinia), in the class of Hexacorallia. Representative species of various genera are noted in brackets. The cladogram is based on the NCBI taxonomy using Common Tree. The ggtree R package was used for visualization [36]. (B) Commonly used laboratory Aiptasia strains CC7, F003, and H2 with their respective algal symbiont assemblages and reported ITS2 type assignments. Note that *Symbiodinium linucheae *is no longer considered valid under the International Code of Nomenclature (ICN) for Algae, Fungi, and Plants because the type specimen was a living culture rather than a permanent deposition [37]. Photo credits: Melanie Dörr. (C) GC-proportion square-binned blob plot (horizontal axis = GC content, vertical axis = sequence coverage) confirming the absence of putative contamination with contigs from other species. Blob sizes are proportional to scaffold lengths and colored by phylum (light blue = Cnidaria, dark blue = no hit). (D) Genome assembly of Aiptasia strain F003. The BlobToolKit v4.1.2 snailplot shows key assembly metrics and BUSCO gene completeness. The main plot is divided into 1,000 size-ordered bins around the circumference. Each bin represents 0.1% of the 237.34 Mb assembly. The central light grey spiral represents the cumulative scaffold count on a logarithmic scale. The dark grey spiral shows the distribution of scaffold lengths with the plot radius scaled to the longest scaffold (664 kb, red). The dark orange and light orange arcs indicate N50 (77.93 kb) and N90 (9.18 kb) scaffold lengths, respectively (also noted in the top left corner). The dark and light blue outer rings represent the GC (36.50%), AT (63.50%), and N (0%) composition (as displayed in the bottom right corner). The top right corner summarizes complete, fragmented, duplicated, and missing BUSCO genes (based on metazoa_odb10, v5.8.2, *n* = 954). The bottom left corner indicates the scale of the genome assembly (~237 Mb) and its longest scaffold (664 kb). (E) Repeat content of the Aiptasia strain F003 genome. A total of 78.07 Mb (32.89%) of the genome assembly consists of repeat elements. Unclassified repeats make up 43.18 Mb (18.19%) of the genome, while transposable elements make up 20.14 Mb (8.49%).

*Exaiptasia diaphana *(Eukaryota; Metazoa; Eumetazoa; Cnidaria; Anthozoa; Hexacorallia; Actiniaria; Aiptasiidae; *Exaiptasia*; NCBI:txid2652724), commonly Aiptasia, is a species of actiniarian sea anemones in the genus *Exaiptasia*, closely related to major reef-building stony corals (Scleractinia) within the class Hexacorallia in the cnidarian subphylum Anthozoa (Figure 1A). Several *Exaiptasia diaphana* strains are used in laboratories worldwide (Figure 1B), including H2 [1] originally obtained from Hawaii, F003 [2], and CC7 [3] originally collected in North Carolina, as well as strains from the Great Barrier Reef (GBR) [4], New Zealand [5], and the Red Sea [6].

Just like reef-building corals, Aiptasia live in symbiosis with photosynthetic dinoflagellate algae (family Symbiodiniaceae) [7, 8] and other microbial partners [9]. Algal symbiont associations vary between different Aiptasia strains, with strain F003 (female) hosting a mixed Symbiodiniaceae assemblage of *Breviolum minutum* (SSB01, majority ITS2 type sequence B1) and *Symbiodinium linucheae* (SSA01, majority ITS2 type sequence A4), whereas H2 (female) and CC7 (male) anemones form single-symbiont associations with SSB01 and SSA01, respectively [1, 2] (Figure 1B).

The endosymbiosis of dinoflagellates with hexacorallians, including reef-building stony corals and sea anemones, is vulnerable to rising sea temperatures and ocean acidification [10]. Coral bleaching, i.e., the loss of algal symbionts [11], is one of the main drivers of coral mortality and reef degradation [12]. Coral responses to environmental stress are also shaped by diverse other microbial partners that contribute to host physiology, nutrient cycling, and stress tolerance [13, 14]. In particular, shifts in bacterial community composition have been recorded under thermal stress, and experimental microbiome manipulation has shown that beneficial bacteria can improve bleaching resilience and recovery in corals [15, 16]. Yet, we lack a general understanding of the underlying molecular mechanisms [16]. This is, at least in part, due to the fact that coral research remains challenging: the endangered status of many coral species makes sampling prohibitive, long generation times complicate long-term (genetic) studies, and calcareous exoskeletons interfere with experimental manipulation [17].

To address these challenges, Aiptasia sea anemones have been used to study cnidarian-dinoflagellate symbiosis with published work dating back at least to the 1970s [7, 18]. Unlike reef-building corals, which are colonial animals, Aiptasia are solitary, single-polyp anemones. They (i) are comparatively small, fast-growing, and easy to maintain under laboratory conditions, (ii) provide access to effectively unlimited numbers of clonal individuals, (iii) can be maintained in non-symbiotic (aposymbiotic or axenic) states (facultative symbiosis) [19], and (iv) are amenable to microscopic, molecular, and genetic manipulation [20, 21]. Aiptasia thus enables simple maintenance, high-throughput experimentation under controlled laboratory conditions, and the disassembly and reassembly of defined holobiont configurations [19, 22]. Consequently, Aiptasia sea anemones have grown into a powerful model poised to advance our understanding of the functional and mechanistic aspects underlying cnidarian-algal-microbiome interactions.

Recent genome sequencing efforts across Anthozoa continue to expand species-specific genomic resources for comparative and mechanistic studies of coral biology, including biomineralization, environmental adaptation, and resilience [23–25]. Although genomic and transcriptomic resources are available for Aiptasia [26, 27], strain-specific genome assemblies remain limited. Of the commonly used laboratory strains CC7, H2, and F003, only CC7 has a published genome [28]. To support the increasing use of Aiptasia strains F003 and H2 in bacterial community analyses, thermal stress assays, and metagenomic sequencing [29, 30], we provide the first genome sequence of *Exaiptasia diaphana* strain F003 to complement existing genomic resources and facilitate future holobiont research.

## Results and discussion

### Genome assembly and repeat elements identification

The genome of *Exaiptasia diaphana* strain F003 was sequenced using one aposymbiotic adult polyp collected under long-term rearing conditions at the University of Konstanz. Genomic DNA was sequenced on an Oxford Nanopore Technologies (ONT) MinION platform using a FLO-MIN114.012 flow cell, generating 7,636,000 raw, unfiltered reads with a mean read length of 1,084.1 base pairs (bp) and a mean Phred-scaled quality score (Q score) of 15.4 (97.12% base-call accuracy). A total of 7,628,266 cleaned reads remained after adapter trimming with Porechop v0.2.4. Reads containing internal adapter sequences were treated as putative chimeras and discarded using the --discard_middle option. An initial 258 Mb assembly was decontaminated, and a quality assessment of the genome assembly confirmed a phylum-specific assembly and the effective removal of non-cnidarian sequences (Figure 1C). Taxonomic classification of the removed contigs (*n* = 121) during the decontamination step showed that the majority were assigned to Pseudomonadota bacteria, with a smaller number assigned to Annelida, Mollusca, Echinodermata, Arthropoda, and Porifera (Data S1, Data S2). The final nuclear genome assembly spanned 237.34 Mb across 12,480 contigs with a GC content of 36.50% (Figure 1D). The assembly size is consistent with and similar to the *Exaiptasia diaphana* strain CC7 genome, which spans 237 Mb [28]. The here-presented draft assembly is version 1 (V1.0) of Aiptasia strain F003 and was generated from ONT long-read data alone. Although Hi-C data were not available for this study, chromosome-scale scaffolding represents a meaningful future upgrade toward a V2.0 assembly. The current assembly has a contig N50 of 76.47 kb. Although the dataset provided approximately 35× nominal coverage, the ONT reads had a read-length N50 of only 1.83 kb (maximum read length 428 kb). This relatively short read-length distribution likely limited the number of reads capable of spanning long or complex repetitive regions and was therefore probably a major constraint on assembly contiguity, consistent with prior benchmarking of ONT assemblies [31]. Despite the modest contig N50, the assembly retained high gene-space completeness (96.50% complete BUSCOs, see below), indicating that read length primarily constrained contiguity rather than overall genome recovery. Contigs were joined using Flye's internal --scaffold function, which uses the assembly repeat graph together with the existing read alignments to connect contigs where the graph supports an unambiguous path across a gap. This procedure resulted in 12,423 scaffolds with a scaffold N50 of 77.93 kb. The longest scaffold spanned 664.23 kb (Table 1). The genome assembly is highly complete with a BUSCO completeness of 96.50% (single = 94.80%, duplicated = 1.70%, fragmented = 1.70%, and missing = 1.80% genes) based on the metazoa_odb10 reference dataset (Table 1, Figure 1D). Repetitive sequences identified through a combined approach employing RepeatModeler and Extensive *de novo* TE Annotator (EDTA) comprised a total of 78.07 Mb (32.89%) of the assembly. The fraction is slightly higher than the fraction of repetitive sequences (26%) reported in the Aiptasia strain CC7 genome [28]. In the Aiptasia strain F003 assembly, unclassified repeats comprised 43.18 Mb (18.19%) of the genome, transposable elements made up 20.14 Mb (8.49%), and retroelements accounted for 10.42 Mb (4.39%) (Figure 1E, Table S1).

### Mitogenome assembly

The mitochondrial genome was assembled into a single contig of size 19.79 kb (Table 1), consistent with the circular mitochondrial genomes typically reported for actiniarian sea anemones [32]. Anthozoan actiniarian sea anemones are known for their variable mitogenome structure, with sizes varying from 16 kb (e.g., *Nematostella* sp.) to over 20 kb (e.g., *Urticina eques*). Interestingly, although it has been reported that GC content across nuclear loci in Hexacorallia is significantly higher than across mitochondrial loci [33], the Aiptasia mitogenome GC content (37.57%) was 1.07% higher compared to the nuclear genome assembly (36.50%) (Table 1). Other closely related taxa, such as the Zoantharia, have also been reported to exhibit slightly higher (0.5%) mitochondrial genome GC contents [33], and anthozoan mitogenomes are generally known to have higher GC content than medusozoans [34].

**Table 1:** Genome assembly and functional annotation statistics of *Exaiptasia diaphana *(Aiptasia) strain F003. The percentage of functionally annotated proteins refers to the proportion of predicted proteins (aa) for which at least one functional annotation was successfully retrieved.

Genome assembly data	
**Project accession data**	
Assembly identifier	AiptasiaF003_V1
Assembly date	2024-03-01
Species	*Exaiptasia diaphana*
Strain	F003
Specimen	Aiptasia F003
NCBI taxonomy ID	2652724
ToLID	jaExaDiap
BioProject ID	PRJNA1089063
BioSample IDs	SAMN40517710 (genome) SAMN46427288 (RNA-seq)
Isolate information	Aiptasia F003 aposymbiotic
**Raw data accessions**	
DNA MinION	SRR28385942
RNA MinION	SRR32136522
**Genome assembly**	
Assembly accession number	JBWBEE000000000
Assembled genome size (Mb)	237.34
Number of contigs	12,480
Contig N50 length (kb)	76.47
Number of scaffolds	12,423
Scaffold N50 length (kb)	77.93
Longest scaffold (kb)	664.23
GC content (%)	36.50
Repeat content (%)	32.89
**BUSCO (metazoa_odb10, v5.8.2, *n* = 954)**	
Completeness (%)	96.50
- Single-copy (%)	94.80
- Duplicated (%)	1.70
Fragmented (%)	1.70
Missing (%)	1.80
**Single-contig mitochondrial genome assembly**	
Assembled mitochondrial genome size (kb)	19.79
GC content (%)	37.57
**Gene prediction and functional annotation**	
Number of protein-coding genes	29,589
- Number of single-exon genes	5,267 (~17.80% of genes)
- Number of pseudogenes	2
Number of mRNAs	30,323
Number of tRNAs	802 (488 eukaryotic high confidence set)
Functionally annotated proteins (%)	85.00

### Gene prediction and functional annotation

To assist gene identification and annotation, total RNA of aposymbiotic F003 Aiptasia polyps was sequenced using the ONT MinION platform. A total of 29,589 protein-coding genes (including 2 pseudogenes) were identified, of which 27,152 (91.76%) represented complete gene models with start and stop codons (Table 1 and Table S2). Overall, 85.00% of the predicted protein-coding genes had functional annotations (Table 1). Here, the annotation rate refers to the proportion of predicted proteins for which at least one functional annotation was successfully retrieved. Functional annotations were assigned by comparing translated protein sequences to known protein families, conserved domains, and orthologous groups using tools such as InterProScan, eggNOG-mapper, and COGs (Table S2). As for the genome size, the number of genes is very similar to the *Exaiptasia diaphana* strain CC7 genome, which totals 29,269 genes [28]. Comparative orthology analysis using OrthoVenn3 [35] identified 16,548 orthologous groups (OGs), corresponding to 17,867 shared protein sequences between F003 and CC7, including 10,493 single-copy orthologs. F003 contained more strain-specific OGs (822) than CC7 (497), suggesting greater gene repertoire expansion in F003 (Figure S1). The F003 strain-specific gene models identified through the OrthoVenn3 orthology analysis together with their orthogroup assignments and available functional annotations are provided in the Supplement (Data S3).

## Materials and methods

### Aiptasia rearing and bleaching

Symbiotic anemones were reared in artificial seawater (ASW; PRO-REEF Sea Salt, Tropic Marin) at 35 g L^−1^ salinity and 25 °C with a 12-hour light, 12-hour dark cycle (~70–80 µmol photons m^−2 ^s^−1^ light intensity). Animals were fed once per week with freshly hatched *Artemia* nauplii (Ocean Nutrition). One day after feeding, anemone tanks were cleaned, and rearing water was exchanged. Aposymbiotic Aiptasia polyps of the clonal strain F003 were generated using a menthol/diuron treatment as previously described [38]. Briefly, menthol (20% w/v in ethanol) was added to 1 µm-filtered ASW to a final concentration of 0.19 mmol L^−1^. The anemones were incubated in the menthol/ASW solution for 8 hours during the 12-hour light period. For the following 16 hours, the anemones were incubated in a fresh solution of 1 µm-filtered ASW containing diuron (DCMU) (100 mM in ethanol) at a final concentration of 5 µM L^−1^ to inhibit the re-establishment of the algal symbiosis. The 24-hour treatment was repeated for four consecutive days, followed by a three-day break during which the anemones were kept in fresh ASW. During the seven-day treatment, the anemones were maintained under rearing conditions. The seven-day treatment cycle was repeated twice, after which the anemones were kept in darkness until sampling and subsequent nucleic acid extraction. Successful bleaching, i.e., expulsion of Symbiodiniaceae, was confirmed using a Zeiss Stemi 2000-C stereomicroscope equipped with a fluorescence green longpass (LP) filter adapter (excitation 510–540 nm, emission 600 nm LP) and UV light illuminator (NIGHTSEA).

### DNA extraction and sequencing

DNA extraction was conducted at the Sequencing Analysis Core Facility (SequAna) at the University of Konstanz. One aposymbiotic Aiptasia anemone of the clonal strain F003 was rinsed in 1× PBS and homogenized using a Polytron PT 1200 E homogenizer (Kinematica, Switzerland). DNA was extracted using the DNeasy Blood & Tissue Kit (Qiagen, Hilden, Germany) according to the manufacturer’s instructions. The Qubit dsDNA High Sensitivity Assay Kit (Thermo Fisher Scientific, Waltham, Massachusetts, USA) was used to assess DNA quantity. The DNA library was prepared with 300 fmol of gDNA following the manufacturer’s protocol for the Ligation Sequencing Kit V14 (SQK - LSK114, Oxford Nanopore Technologies, Oxford, UK). DNA sequencing was performed using the Oxford Nanopore Technologies (ONT) MinION Mk1B platform (Oxford Nanopore Technologies, Oxford, UK) with a FLO-MIN114.012 flow cell and super-accurate base-calling (dna_r10.4.1_e8.2_400bps_sup@v4.2.0). The flow cell was run for 72 h following the standard manufacturer’s protocol.

### RNA extraction and sequencing

RNA extraction was conducted at the Sequencing Analysis Core Facility (SequAna) at the University of Konstanz. Total RNA was extracted from 2 pools of 5 aposymbiotic F003 Aiptasia anemones using the RNeasy Mini Kit (Qiagen, Hilden, Germany). First, animals were lysed and homogenized in 600 µL Buffer RLT using a Polytron PT 1200 E homogenizer (Kinematica, Switzerland). Next, total RNA was extracted with an additional on-column DNAse I digestion (RNase-Free DNase Set, Qiagen, Hilden, Germany) according to the manufacturer's instructions. Both RNA extractions were concentrated via lithium chloride precipitation. Briefly, an equal volume of 7.5 M lithium chloride was added to each sample, and the samples were incubated overnight at −20 °C. After centrifugation (12,000 × g, 15 min), the supernatant was removed. The pellets were washed with 2.5 sample volumes of 70% ethanol, centrifuged (12,000 × g, 2 min), air-dried (30 min), and dissolved in RNase-free water. RNA quantity and quality were assessed using the Qubit RNA High Sensitivity Assay Kit (Thermo Fisher Scientific, Waltham, Massachusetts, USA), NanoDrop spectrophotometry, and 1% agarose gel electrophoresis. The two RNA extractions were pooled into a single sample, and a total RNA-seq library was constructed using the Direct RNA Sequencing Kit (SQK-RNA004, Oxford Nanopore Technologies, Oxford, UK) according to the manufacturer’s protocol. Direct RNA sequencing was performed on the ONT MinION platform using a FLO-MIN004RA flow cell and super-accurate base-calling (rna004_130bps_sup@v3.0.1). As for DNA sequencing, the flow cell was run for 72 h following the standard manufacturer’s protocol.

### Nuclear and mitochondrial genome assembly and evaluation

Adapters were trimmed from the ONT DNA sequencing reads using Porechop v0.2.4 (RRID:SCR_016967), and reads were assembled using NECAT v0.0.1 (RRID:SCR_025350) [39], CANU v2.2 (RRID:SCR_015880) [40], and Flye v2.9.3 (RRID:SCR_017016) [41] (Table S3). NECAT and Canu incorporate read-correction procedures into their assembly workflows through progressive two-step correction and adaptive k-mer-weighted overlap correction, respectively. In contrast, Flye constructs a repeat graph directly from uncorrected reads and performs assembly consensus polishing of the resulting assembly using read alignments. Flye was run with --genome-size 275m and --scaffold; all other parameters were left at their default settings. The Flye assembly was chosen for downstream analysis since it showed the best assembly statistics [42]. Genome assembly characteristics were evaluated and visualized as taxon-annotated Guanine–Cytosine (GC)-proportion plots using BlobToolKit v4.1.2 (RRID:SCR_025882) [43]. The putative taxonomic identity of contigs was determined with searches against the National Center for Biotechnology Information (NCBI) non-redundant nucleotide database using BLASTn v2.14.1 (RRID:SCR_001598) and the UniProt database with DIAMOND v2.1.8 (RRID:SCR_016071) [44], following the BlobToolKit instructions. The initial assembly was decontaminated by removing non-cnidarian contigs based on GC content, coverage, and taxonomic assignment information provided by BlobToolKit. Completeness and contiguity of the assembly were evaluated using BUSCO v5.8.2 (RRID:SCR_015008) [45], whereby the Archaea and Bacteria datasets were used to assess decontamination while the Eukaryota and Metazoa OrthoDB v10 datasets were used to assess completeness across a broad evolutionary range. The mitochondrial genome was assembled using GetOrganelle v1.7.7.1 (RRID:SCR_022963) [46], and MITOS v2.1.0 [47] was used to obtain functional annotations of the mitogenomic sequence.

### DNA repeat identification

Genomic DNA repeats were identified using a combination of RepeatModeler v2.0.4 (RRID:SCR_015027) [48] and the Extensive *de novo* TE Annotator (EDTA) v2.2.0 (RRID:SCR_022063) [49] (Table S3). Identified repeats were masked with RepeatMasker v4.1.6 (RRID:SCR_012954) [50] using custom *Exaiptasia*-specific repeat libraries under the "LTRStruct'' option, while default settings were applied for all other parameters**. **To visualize the hierarchical distribution of repeat elements, we utilized a sunburst plot using the Plotly Express library in Python (Plotly Technologies Inc.).

### Gene prediction and functional annotation

All gene prediction and functional annotation steps were orchestrated using our fully automated pipeline, GeneForge v1.0 [51], which supports both BRAKER v3.0.8 (RRID:SCR_018964) [52] and funannotate v1.8.15 (RRID:SCR_023039) [53]. GeneForge evaluated the completeness of the gene set annotations generated by BRAKER and funannotate using BUSCO, based on the corresponding predicted protein sequences. The gene models predicted using funannotate achieved the highest BUSCO completeness and were therefore selected for all downstream analyses (Table S3). As part of funannotate v1.8.15 (RRID:SCR_023039), five ab initio predictors were run independently: AUGUSTUS (RRID:SCR_008417), SNAP (RRID:SCR_007936 ), glimmerHMM (RRID:SCR_002654), CodingQuarry, and GeneMark-ES/ET [54–59]. Their outputs were then compiled into a single consensus gene set using EVidenceModeler (EVM) (RRID:SCR_014659) [60], which weights each ab initio prediction against the aligned RNA-seq evidence (see below) to resolve conflicting gene models. RNA-seq evidence came from two sources and served distinct roles. ONT direct RNA sequencing of aposymbiotic F003 polyps generated in this study was trimmed using Porechop v0.2.4 (RRID:SCR_016967) (Table 3), aligned to the assembled genome using STAR v2.7.11b (RRID:SCR_004463) [61], and assembled with StringTie v2.2.1 (RRID:SCR_016323) [62]. The resulting long-read transcript models were used to extend UTR boundaries and support splice sites during EVM weighting. Published Illumina RNA-seq data from aposymbiotic Aiptasia larval cells [63] provided complementary, independent short-read transcript evidence for gene models with limited or no support from the ONT dataset. All predicted proteins were functionally annotated using a combination of databases and tools. General functional annotation was performed with InterProScan v5.72-103.0 (RRID:SCR_005829) [64], eggNOG-mapper v2.1.12 (RRID:SCR_021165) [65, 66], BUSCO v5.8.2 (RRID:SCR_015008) [45], Pfam (RRID:SCR_004726) [67], and COGs (RRID:SCR_007139). Carbohydrate-active enzyme families were identified using the CAZy database (RRID:SCR_012909) [68], while proteases were classified using MEROPS (RRID:SCR_007777). Moreover, we searched the NCBI and UniProtKB/SwissProt databases using BLASTp v2.14.1 (RRID:SCR_001010) and DIAMOND v2.1.8 (RRID:SCR_016071) for gene names and gene product descriptions [44, 69]. Lastly, we predicted transmembrane topology and signal peptides using Phobius v1.01 (RRID:SCR_015643) and SignalP v6.0 (RRID:SCR_015644) [70, 71].

## Availability of source code and requirements

• Project name: Aiptasia_F003_genome
• Project homepage: https://github.com/SequAna-Ukon/Aiptasia_F003_genome
• Operating system: Linux
• Programming language: Bash
• Other requirements: The analysis pipelines require the bioinformatic tools and software dependencies listed in Table S3. Detailed installation instructions, pipeline descriptions, and commands are provided in the GitHub repository
• License: MIT license

## Data Availability

Sequencing data and genome assembly (genome assembly ID: GCA_056151815.1) are available under NCBI BioProject ID PRJNA1089063. The genome assembly FASTA file, coding gene annotation GFF file, coding gene nucleotide and protein sequence FASTA files, repeat/transposable element annotation GFF file, and BUSCO output files are available through Zenodo [72].

## References

[1] Xiang T, Hambleton EA, DeNofrio JC Isolation of clonal axenic strains of the symbiotic dinoflagellate *Symbiodinium* and their growth and host specificity. J. Phycol., 2013; 49(3):447–458. doi:10.1111/jpy.12055. 27007034

[2] Grawunder D, Hambleton EA, Bucher M Induction of Gametogenesis in the Cnidarian Endosymbiosis Model *Aiptasia* sp. Sci. Rep., 2015; 5(1):15677. doi:10.1038/srep15677. PMC4620495 26498008

[3] Sunagawa S, Wilson EC, Thaler M Generation and analysis of transcriptomic resources for a model system on the rise: the sea anemone Aiptasia pallida and its dinoflagellate endosymbiont. BMC Genom., 2009; 10(1):258. doi:10.1186/1471-2164-10-258. PMC2702317 19500365

[4] Dungan AM, Hartman LM, Tortorelli G Exaiptasia diaphana from the great barrier reef: a valuable resource for coral symbiosis research. Symbiosis, 2020; 80(2):195–206. doi:10.1007/s13199-020-00665-0.

[5] Matthews JL, Crowder CM, Oakley CA Optimal nutrient exchange and immune responses operate in partner specificity in the cnidarian-dinoflagellate symbiosis. Proc. Natl. Acad. Sci. USA, 2017; 114(50):13194–13199. doi:10.1073/pnas.1710733114. PMC5740609 29158383

[6] Ahmed HI, Herrera M, Liew YJ Long-Term Temperature Stress in the Coral Model Aiptasia Supports the “Anna Karenina Principle” for Bacterial Microbiomes. Front. Microbiol., 2019; 10: 975. doi:10.3389/fmicb.2019.00975. PMC6517863 31139158

[7] Glider WV, Phipps DW, Pardy RL. Localization of symbiotic dinoflagellate cells within tentacle tissue of *Aiptasia pallida* (Coelenterata, Anthozoa). Trans. Am. Microsc. Soc., 1980; 99(4):426–438. doi:10.2307/3225653.

[8] LaJeunesse TC, Parkinson JE, Gabrielson PW Systematic revision of symbiodiniaceae highlights the antiquity and diversity of coral endosymbionts. Curr. Biol., 2018; 28(16):2570–2580. doi:10.1016/j.cub.2018.07.008. 30100341

[9] Röthig T, Costa RM, Simona F Distinct bacterial communities associated with the coral model *Aiptasia* in Aposymbiotic and symbiotic states with *Symbiodinium* . Front. Mar. Sci., 2016; 3: 234. doi:10.3389/fmars.2016.00234.

[10] Hoegh-Guldberg O. Climate change, coral bleaching and the future of the world’s coral reefs. Mar. Freshw. Res., 1999; 50(8):839–866. doi:10.1071/mf99078.

[11] Lesser MP. Oxidative stress in marine environments: biochemistry and physiological ecology. Annu. Rev. Physiol., 2006; 68(1):253–278. doi:10.1146/annurev.physiol.68.040104.110001. 16460273

[12] Hughes TP, Kerry JT, Álvarez-Noriega M Global warming and recurrent mass bleaching of corals. Nature, 2017; 543(7645):373–377. doi:10.1038/nature21707. 28300113

[13] Voolstra CR, Raina JB, Dörr M The coral microbiome in sickness, in health and in a changing world. Nat. Rev. Microbiol., 2024; 22(8):460–475. doi:10.1038/s41579-024-01015-3. 38438489

[14] Bourne DG, Morrow KM, Webster NS. Insights into the coral microbiome: Underpinning the health and resilience of reef ecosystems. Annu. Rev. Microbiol., 2016; 70(1):317–340. doi:10.1146/annurev-micro-102215-095440. 27482741

[15] Ziegler M, Seneca FO, Yum LK Bacterial community dynamics are linked to patterns of coral heat tolerance. Nat. Commun., 2017; 8(1):14213. doi:10.1038/ncomms14213. PMC5309854 28186132

[16] Dörr M, Barno AR, Villela H Microbial-based therapies to restore and rehabilitate disrupted coral health. In: Coral Reefs of the World. Cham: Springer Nature. doi:10.1007/978-3-031-76692-3_13.

[17] Weis VM, Davy SK, Hoegh-Guldberg O Cell biology in model systems as the key to understanding corals. Trends Ecol. Evol., 2008; 23(7):369–376. doi:10.1016/j.tree.2008.03.004. 18501991

[18] Cook CB. Transfer of 35S-labeled material from food ingested by Aiptasia sp. to its endosymbiotic zooxanthellae. In: Experimental Coelenterate Biology. Honolulu: University of Hawaii Press, 1971; pp. 218–224.

[19] Costa RM, Cárdenas A, Loussert-Fonta C Surface topography, bacterial carrying capacity, and the prospect of microbiome manipulation in the sea anemone coral model Aiptasia. Front. Microbiol., 2021; 12: 637834. doi:10.3389/fmicb.2021.637834. PMC8060496 33897642

[20] Jones VAS, Bucher M, Hambleton EA Microinjection to deliver protein, mRNA, and DNA into zygotes of the cnidarian endosymbiosis model *Aiptasia* sp. Sci. Rep., 2018; 8(1):16437. doi:10.1038/s41598-018-34773-1. PMC6219564 30401930

[21] Puntin G, Sweet M, Fraune S Harnessing the power of model organisms to unravel microbial functions in the coral holobiont. Microbiol. Mol. Biol. Rev., 2022; 86(4):e00053-22. doi:10.1128/mmbr.00053-22. PMC9769930 36287022

[22] Bucher M, Wolfowicz I, Voss PA Development and symbiosis establishment in the cnidarian endosymbiosis model *Aiptasia* sp. Sci. Rep., 2016; 6(1):19867. doi:10.1038/srep19867. PMC4726165 26804034

[23] Conn T, Ashey J, Cunning R Genome assembly and annotation of *Acropora pulchra* from Mo’orea French Polynesia. Gigabyte, 2025; gigabyte153. doi:10.46471/gigabyte.153. PMC11985253 40212849

[24] Liang Y, Xu K, Li J The molecular basis of octocoral calcification revealed by genome and skeletal proteome analyses. GigaScience, 2025; 14: giaf031. doi:10.1093/gigascience/giaf031. PMC11959691 40167990

[25] Fiesinger A, Sharaf A, Alderdice R The genome of the reef-building coral *Porites harrisoni* from the southern Persian/Arabian Gulf. Gigabyte, 2026; gigabyte174. doi:10.46471/gigabyte.174. PMC13040556 41929243

[26] Lehnert EM, Burriesci MS, Pringle JR. Developing the anemone *Aiptasia* as a tractable model for cnidarian-dinoflagellate symbiosis: the transcriptome of aposymbiotic *A. pallida* . BMC Genom., 2012; 13(1):271. doi:10.1186/1471-2164-13-271. PMC3427133 22726260

[27] Baumgarten S, Cziesielski MJ, Thomas L Evidence for miRNA‐mediated modulation of the host transcriptome in cnidarian–dinoflagellate symbiosis. Mol. Ecol., 2017; 27(2):403–418. doi:10.1111/mec.14452. 29218749

[28] Baumgarten S, Simakov O, Esherick LY The genome of *Aiptasia*, a sea anemone model for coral symbiosis. Proc. Natl. Acad. Sci. USA, 2015. 112(38):pp.11893–11898. doi:10.1073/pnas.1513318112. PMC4586855 26324906

[29] Dörr M, Denger J, Maier CS Short-term heat stress assays resolve effects of host strain, repeat stress, and bacterial inoculation on Aiptasia thermal tolerance phenotypes. Coral Reefs, 2023; 42(6):1271–1281. doi:10.1007/s00338-023-02427-y.

[30] Voolstra CR, Colin L, Dörr MS DNA preservation & DNA extraction protocol for field collection of coral samples suitable for host-, marker gene-, and metagenomics-based sequencing approaches. Zenodo. 2025. 10.5281/ZENODO.15200217.

[31] Cosma BM, Shirali Hossein Zade R, Jordan EN Evaluating long-read de novo assembly tools for eukaryotic genomes: insights and considerations. GigaScience, 2022; 12: giad100. doi:10.1093/gigascience/giad100. PMC10673639 38000912

[32] Emblem Å, Okkenhaug S, Weiss ES Sea anemones possess dynamic mitogenome structures. Mol. Phylogenet. Evol., 2014; 75: 184–193. doi:10.1016/j.ympev.2014.02.016. 24613805

[33] Quattrini AM, Snyder KE, Purow-Ruderman R Mito-nuclear discordance within Anthozoa, with notes on unique properties of their mitochondrial genomes. Sci Rep., 2023; 13(1):7443. doi:10.1038/s41598-023-34059-1. PMC10167242 37156831

[34] Feng H, Lv S, Li R Mitochondrial genome comparison reveals the evolution of cnidarians. Ecol. Evol., 2023; 13(6):e10157. doi:10.1002/ece3.10157. PMC10261974 37325715

[35] Sun J, Lu F, Luo Y OrthoVenn3: an integrated platform for exploring and visualizing orthologous data across genomes. Nucleic Acids Res., 2023; 51(W1):397–403. doi:10.1093/nar/gkad313. PMC10320085 37114999

[36] Yu G, Smith DK, Zhu H GGTREE: An r package for visualization and annotation of phylogenetic trees with their covariates and other associated data. Methods Ecol. Evol., 2017; 8(1):28–36. doi:10.1111/2041-210x.12628.

[37] Parkinson JE, Peixoto RS, Symbiodiniaceae VCR. Coral Reefs of the World. Cham: Springer Nature. doi:10.1007/978-3-031-76692-3_2.

[38] Matthews JL, Sproles AE, Oakley CA Menthol-induced bleaching rapidly and effectively provides experimental aposymbiotic sea anemones (*Aiptasia* sp.) for symbiosis investigations. J. Exp. Biol., 2016; 219(3):306–310. doi:10.1242/jeb.128934. 26596538

[39] Chen Y, Nie F, Xie SQ Efficient assembly of nanopore reads via highly accurate and intact error correction. Nat. Commun., 2021; 12(1):60. doi:10.1038/s41467-020-20236-7. PMC7782737 33397900

[40] Koren S, Walenz BP, Berlin K Canu: scalable and accurate long-read assembly via adaptive *k*-mer weighting and repeat separation. Genome Res., 2017; 27(5):722–736. doi:10.1101/gr.215087.116. PMC5411767 28298431

[41] Kolmogorov M, Yuan J, Lin Y Assembly of long, error-prone reads using repeat graphs. Nat. Biotechnol., 2019; 37(5):540–546. doi:10.1038/s41587-019-0072-8. 30936562

[42] Zimin AV, Puiu D, Luo MC Hybrid assembly of the large and highly repetitive genome of *Aegilops tauschii*, a progenitor of bread wheat, with the MaSuRCA mega-reads algorithm. Genome Res., 2017; 27(5):787–792. doi:10.1101/gr.213405.116. PMC5411773 28130360

[43] Challis R, Richards E, Rajan J BlobToolKit – Interactive quality assessment of genome assemblies. G3 (Bethesda), 2020; 10(4):1361–1374. doi:10.1534/g3.119.400908. PMC7144090 32071071

[44] Buchfink B, Reuter K, Drost HG. Sensitive protein alignments at tree-of-life scale using DIAMOND. Nat. Methods, 2021; 18(4):366–368. doi:10.1038/s41592-021-01101-x. PMC8026399 33828273

[45] Simão FA, Waterhouse RM, Ioannidis P BUSCO: assessing genome assembly and annotation completeness with single-copy orthologs. Bioinformatics, 2015; 31(19):3210–3212. doi:10.1093/bioinformatics/btv351. 26059717

[46] Jin JJ, Yu WB, Yang JB GetOrganelle: a fast and versatile toolkit for accurate de novo assembly of organelle genomes. Genome Biol., 2020; 21(1):241. doi:10.1186/s13059-020-02154-5. PMC7488116 32912315

[47] Bernt M, Donath A, Jühling F MITOS: Improved de novo metazoan mitochondrial genome annotation. Mol. Phylogenet. Evol., 2013; 69(2):313–319. doi:10.1016/j.ympev.2012.08.023. 22982435

[48] Flynn JM, Hubley R, Goubert C RepeatModeler2 for automated genomic discovery of transposable element families. Proc. Natl. Acad. Sci. USA, 2020; 117(17):9451–9457. doi:10.1073/pnas.1921046117. PMC7196820 32300014

[49] Ou S, Su W, Liao Y Benchmarking transposable element annotation methods for creation of a streamlined, comprehensive pipeline. Genome Biol., 2019; 20(1):275. doi:10.1186/s13059-019-1905-y. PMC6913007 31843001

[50] Nishimura D. RepeatMasker. Biotech Softw. Internet Rep., 2000; 1(1–2):36–39. doi:10.1089/152791600319259.

[51] Sharaf A, Voolstra CR. SequAna-Ukon/GeneForge: GeneForge v1.0. Zenodo. 2025. 10.5281/ZENODO.16631467.

[52] Gabriel L, Brůna T, Hoff KJ BRAKER3: Fully automated genome annotation using RNA-seq and protein evidence with GeneMark-ETP, AUGUSTUS, and TSEBRA. Genome Res., 2024; 34(5):769–777. doi:10.1101/gr.278090.123. PMC11216308 38866550

[53] Palmer JM, Stajich JE. Funannotate v1.8.1: Eukaryotic genome annotation (v1.8). Zenodo. 2020. 10.5281/zenodo.1134477.

[54] Chan PP, Lin BY, Mak AJ tRNAscan-SE 2.0: improved detection and functional classification of transfer RNA genes. Nucleic Acids Res, 2021; 49(16):9077–9096. doi:10.1093/nar/gkab688. PMC8450103 34417604

[55] Korf I. Gene finding in novel genomes. BMC Bioinform, 2004; 5(1):59. doi:10.1186/1471-2105-5-59. PMC421630 15144565

[56] Majoros WH, Pertea M, Salzberg SL. TigrScan and GlimmerHMM: two open source *ab initio* eukaryotic gene-finders. Bioinformatics, 2004; 20(16):2878–2879. doi:10.1093/bioinformatics/bth315. 15145805

[57] Testa AC, Hane JK, Ellwood SR CodingQuarry: highly accurate hidden Markov model gene prediction in fungal genomes using RNA-seq transcripts. BMC Genom, 2015; 16(1):170. doi:10.1186/s12864-015-1344-4. PMC4363200 25887563

[58] Brůna T, Lomsadze A, Borodovsky M. GeneMark-EP+: eukaryotic gene prediction with self-training in the space of genes and proteins. NAR Genom Bioinform, 2020; 2(2):lqaa026. doi:10.1093/nargab/lqaa026. PMC7222226 32440658

[59] Keller O, Kollmar M, Stanke M A novel hybrid gene prediction method employing protein multiple sequence alignments. Bioinformatics, 2011; 27(6):757–763. doi:10.1093/bioinformatics/btr010. 21216780

[60] Haas BJ, Salzberg SL, Zhu W Automated eukaryotic gene structure annotation using EVidenceModeler and the Program to Assemble Spliced Alignments. Genome Biol, 2008; 9(1):R7. doi:10.1186/gb-2008-9-1-r7. PMC2395244 18190707

[61] Dobin A, Davis CA, Schlesinger F STAR: ultrafast universal RNA-seq aligner. Bioinformatics, 2012; 29(1):15–21. doi:10.1093/bioinformatics/bts635. PMC3530905 23104886

[62] Pertea M, Pertea GM, Antonescu CM StringTie enables improved reconstruction of a transcriptome from RNA-seq reads. Nat. Biotechnol., 2015; 33(3):290–295. doi:10.1038/nbt.3122. PMC4643835 25690850

[63] Jacobovitz MR, Rupp S, Voss PA Dinoflagellate symbionts escape vomocytosis by host cell immune suppression. Nat. Microbiol., 2021; 6(6):769–782. doi:10.1038/s41564-021-00897-w. PMC7611106 33927382

[64] Jones P, Binns D, Chang HY InterProScan 5: genome-scale protein function classification. Bioinformatics, 2014; 30(9):1236–1240. doi:10.1093/bioinformatics/btu031. PMC3998142 24451626

[65] Huerta-Cepas J, Szklarczyk D, Heller D eggNOG 5.0: a hierarchical, functionally and phylogenetically annotated orthology resource based on 5090 organisms and 2502 viruses. Nucleic Acids Res., 2019; 47(D1):309–314. doi:10.1093/nar/gky1085. PMC6324079 30418610

[66] Cantalapiedra CP, Hernández-Plaza A, Letunic I eggNOG-mapper v2: Functional Annotation, Orthology Assignments, and Domain Prediction at the Metagenomic Scale. Mol. Biol. Evol., 2021; 38(12):5825–5829. doi:10.1093/molbev/msab293. PMC8662613 34597405

[67] Finn RD, Bateman A, Clements J Pfam: the protein families database. Nucleic Acids Res, 2013; 42(D1):D222–D230. doi:10.1093/nar/gkt1223. PMC3965110 24288371

[68] Drula E, Garron ML, Dogan S The carbohydrate-active enzyme database: functions and literature. Nucleic Acids Res., 2021; 50(D1):571–577. doi:10.1093/nar/gkab1045. PMC8728194 34850161

[69] Altschul SF, Gish W, Miller W Basic local alignment search tool. J. Mol. Biol, 1990; 215(3):403–410. doi:10.1016/s0022-2836(05)80360-2. 2231712

[70] Kall L, Krogh A, Sonnhammer ELL. Advantages of combined transmembrane topology and signal peptide prediction--the Phobius web server. Nucleic Acids Res, 2007; 35: W429–W432. doi:10.1093/nar/gkm256. PMC1933244 17483518

[71] Teufel F, Almagro Armenteros JJ, Johansen AR SignalP 6.0 predicts all five types of signal peptides using protein language models. Nat. Biotechnol, 2022; 40(7):1023–1025. doi:10.1038/s41587-021-01156-3. PMC9287161 34980915

[72] Dörr MS, Sharaf A, Colin L Exaiptasia diaphana Aiptasia F003 V1.0 final. Zenodo. 2026; 10.5281/ZENODO.21918813.

