# Supplementary material for: The genome of the coral model sea anemone Exaiptasia diaphana (Aiptasia) strain F003
Source: GigaByte. 2026 Sep 14;2026:gigabyte188. doi: 10.46471/gigabyte.188 (PMC13613205; doi:10.46471/gigabyte.188)
Supplement: Peer review history

# Reviewer report: Review MS


Reviewer name and names of any other individual's who aided in reviewer Dr. Lukas BeckerDo you understand and agree to our policy of having open and named reviews, and having your review included with the published papers. (If no, please inform the editor that you cannot review this manuscript.)YesIs the language of sufficient quality?YesPlease add additional comments on language quality to clarify if needed
Line 65-66: coral should be written in plural: corals - I would use the following beginning: Unlike reef building corals, ...Are all data available and do they match the descriptions in the paper? YesAdditional CommentsAre the data and metadata consistent with relevant minimum information or reporting standards? See GigaDB checklists for examples <a href="http://gigadb.org/site/guide" target="_blank">http://gigadb.org/site/guide</a>YesAdditional CommentsThe Zenodo repository contains all information required to evaluate and reproduce the bioinformatic pipeline. The scripts are clearly documented, and the NCBI BioProject and associated FTP site provide all genomic data necessary to access, assess, and reuse this genomic resource.Is the data acquisition clear, complete and methodologically sound?NoAdditional CommentsPlease clarify the phrase “filtering for chimeric adapters.” Based on the reported Porechop v0.2.4 command using the --discard_middle option, it appears that reads containing internal adapter sequences were treated as putative chimeric reads and discarded. The wording should therefore be revised to distinguish between internal adapter sequences and chimeric reads.Is there sufficient detail in the methods and data-processing steps to allow reproduction?NoAdditional CommentsAt line 231, the authors state that Flye was run with default parameters. However, the reported command includes the explicit options --genome-size 275m and --scaffold. Please revise the Methods to mention these settings and avoid describing the analysis as having been performed entirely with default parameters.Is there sufficient data validation and statistical analyses of data quality? YesAdditional CommentsIs the validation suitable for this type of data?YesAdditional CommentsIs there sufficient information for others to reuse this dataset or integrate it with other data?YesAdditional CommentsAny Additional Overall Comments to the AuthorAs the animal was maintained under aposymbiotic conditions, it could be informative to provide a brief summary of the BlobToolKit taxonomic assignments, particularly the taxa represented among the sequences removed during decontamination. Given that the assembly size was reduced from approximately 258 Mb to 237.34 Mb, the excluded contigs may contain useful information on residual symbionts, associated microorganisms, or other non-target sequences. Although not essential for the Data Release, the authors could consider providing the filtered contigs, together with their taxonomic assignments, as a supplementary file or in a public repository to facilitate transparency and future reuse. Although not essential for this Data Release, the manuscript could be further enhanced by providing a supplementary table listing the 325 genes predicted only in one strain, together with their orthogroup assignments and available functional annotations. This would facilitate reuse of the dataset and provide a useful starting point for future comparative analyses. The gene prediction and functional annotation are highly comprehensive and well executed, providing a valuable and thoroughly characterized genomic resource. I identified only two minor points that require clarification in the Methods: the wording concerning the removal of putative chimeric reads with Porechop and the description of the Flye assembly parameters. The remaining comments are optional suggestions that could improve transparency and facilitate future reuse of the dataset, but they are not essential for publication.RecommendationMinor Revision


# Reviewer report: Review MS


Reviewer name and names of any other individual's who aided in reviewer chao bianDo you understand and agree to our policy of having open and named reviews, and having your review included with the published papers. (If no, please inform the editor that you cannot review this manuscript.)YesIs the language of sufficient quality?YesPlease add additional comments on language quality to clarify if needed
Are all data available and do they match the descriptions in the paper? YesAdditional CommentsAre the data and metadata consistent with relevant minimum information or reporting standards? See GigaDB checklists for examples <a href="http://gigadb.org/site/guide" target="_blank">http://gigadb.org/site/guide</a>YesAdditional CommentsIs the data acquisition clear, complete and methodologically sound?YesAdditional CommentsIs there sufficient detail in the methods and data-processing steps to allow reproduction?YesAdditional CommentsIs there sufficient data validation and statistical analyses of data quality? YesAdditional CommentsIs the validation suitable for this type of data?YesAdditional CommentsIs there sufficient information for others to reuse this dataset or integrate it with other data?YesAdditional CommentsThis study, entitled “The genome of the coral model sea anemone Exaiptasia diaphana (Aiptasia) strain F003”, reports a genome assembly of sea anemone. This assembly was used the ONT sequencing for assembling. The N50 of contigs and scaffolds are 76.47kb and 77.93kb. 29589 genes were identified. For the quality of this assembly, why did not use the hifiasm to assemble the sequences? These contigs are indeed fragment. Pure ONT sequencing will largely improve the extensibility of contigs. This fragmented contigs have large potential to be of high quality. In addition, the author may sequence the HIC data to cluster this assembly to be a chromosome genome. Annotation pipeline is also somewhat outdate. Braker2 or egapx(NCBI) are indeed recommended. How many BUSCO value for the protein set?Any Additional Overall Comments to the AuthorThis study, entitled “The genome of the coral model sea anemone Exaiptasia diaphana (Aiptasia) strain F003”, reports a genome assembly of sea anemone. This assembly was used the ONT sequencing for assembling. The N50 of contigs and scaffolds are 76.47kb and 77.93kb. 29589 genes were identified. For the quality of this assembly, why did not use the hifiasm to assemble the sequences? These contigs are indeed fragment. Pure ONT sequencing will largely improve the extensibility of contigs. This fragmented contigs have large potential to be of high quality. In addition, the author may sequence the HIC data to cluster this assembly to be a chromosome genome. Annotation pipeline is also somewhat outdate. Braker2 or egapx(NCBI) are indeed recommended. How many BUSCO value for the protein set?RecommendationMajor Revision


# Reviewer report: Review MS


Reviewer name and names of any other individual's who aided in reviewer Jianbo JianDo you understand and agree to our policy of having open and named reviews, and having your review included with the published papers. (If no, please inform the editor that you cannot review this manuscript.)YesIs the language of sufficient quality?YesPlease add additional comments on language quality to clarify if needed
Are all data available and do they match the descriptions in the paper? YesAdditional CommentsAre the data and metadata consistent with relevant minimum information or reporting standards? See GigaDB checklists for examples <a href="http://gigadb.org/site/guide" target="_blank">http://gigadb.org/site/guide</a>YesAdditional CommentsIs the data acquisition clear, complete and methodologically sound?YesAdditional CommentsIs there sufficient detail in the methods and data-processing steps to allow reproduction?NoAdditional Comments5、Gene prediction appears simple; more details are needed.Is there sufficient data validation and statistical analyses of data quality? YesAdditional CommentsIs the validation suitable for this type of data?YesAdditional CommentsIs there sufficient information for others to reuse this dataset or integrate it with other data?YesAdditional CommentsAny Additional Overall Comments to the AuthorThe authors reported the genome of the coral model sea anemone Exaiptasia diaphana (Aiptasia) strain F003 using Oxford Nanopore Technologies (ONT). The sample F003 is significant. Nevertheless, the genome quality appears to not so well as the contig N50 is only 76.47 kb. I have the following questions. It is hoped that these comments will be beneficial for improving the manuscript. 1、Is the low Contig N50 due to insufficient ONT data or the ONT read lengths not long? 2、The scaffold N50 improved slightly from 76.47 kb to 77.93 kb. Scaffolding relied solely on large-insert-size or Hi-C data; ONT reads were used for assembly, and the same ONT data was reused for scaffolding—potentially introducing errors. 3、ONT data still has high error rates—does genome assembly include error correction? 4、We used TE Annotator (EDTA) v2.2.0 for repeat annotation. Since EDTA is commonly applied to plant genomes, could you clarify why this pipeline was selected? 5、Gene prediction appears simple; more details are needed.RecommendationMajor Revision


# Reviewer report: Re-Review MS


Indicate in the comments box below whether you are happy with the changes made or if the manuscript is unacceptable.Comments on revised manuscriptThe authors have well addressed all my comments. This paper is pretty well now.Indicate in the comments box below whether you are happy with the changes made or if the manuscript is unacceptable.Comments on revised manuscriptThe authors have well addressed all my comments. This paper is pretty well now.


# Reviewer report: Re-Review MS


Indicate in the comments box below whether you are happy with the changes made or if the manuscript is unacceptable.Comments on revised manuscriptReviewer comments have been satisfactorily addressed, and I have no further commentsIndicate in the comments box below whether you are happy with the changes made or if the manuscript is unacceptable.Comments on revised manuscriptReviewer comments have been satisfactorily addressed, and I have no further comments


# Editor report: Accept


Editor’s AssessmentThe manuscript is ready for formal acceptance.Editor’s AssessmentThe manuscript is ready for formal acceptance.
